# Giraffes make decisions based on statistical information

**DOI:** 10.1038/s41598-023-32615-3

**Published:** 2023-05-04

**Authors:** Alvaro L. Caicoya, Montserrat Colell, Federica Amici

**Affiliations:** 1 Department of Clinical Psychology and Psychobiology, Faculty of Psychology, University of Barcelona, Barcelona, Spain; 2 Institute of Neurosciences, University of Barcelona, Barcelona, Spain; 3 Research Group Human Biology and Primate Cognition, Institute of Biology, University of Leipzig, Leipzig, Germany; 4 Department of Comparative Cultural Psychology, Max Planck Institute for Evolutionary Anthropology, Leipzig, Germany

**Keywords:** Zoology, Animal behaviour

## Abstract

The ability to make inferences based on statistical information has so far been tested only in animals having large brains in relation to their body size, like primates and parrots. Here we tested if giraffes (*Giraffa camelopardalis*), despite having a smaller relative brain size, can rely on relative frequencies to predict sampling outcomes. We presented them with two transparent containers filled with different quantities of highly-liked food and less-preferred food. The experimenter covertly drew one piece of food from each container, and let the giraffe choose between the two options. In the first task, we varied the quantity and relative frequency of highly-liked and less-preferred food pieces. In the second task, we inserted a physical barrier in both containers, so giraffes only had to take into account the upper part of the container when predicting the outcome. In both tasks giraffes successfully selected the container more likely to provide the highly-liked food, integrating physical information to correctly predict sampling information. By ruling out alternative explanations based on simpler quantity heuristics and learning processes, we showed that giraffes can make decisions based on statistical inferences.

## Introduction

Reasoning about probabilities has long been considered a complex ability, traditionally ascribed only to adult humans^1–3^. When reasoning about probabilities, individuals deal with a situation of uncertainty in which not all the information is available, and statistically infer which option might lead to the best possible outcome. This kind of decision making is very important in the real world, where only limited information is often available and not all possible outcomes are known with certainty^4^. In the last decade, experimental evidence has shown that statistical inference is not limited to adult humans, but it emerges early on during human development. Twelve-month old infants, for instance, can predict outcomes from a sampling event and make decisions based on the comparison of relative quantities^5^, whereas 4.5-month-olds can even account for the presence of physical constraints that could affect the sampling process^6^. Other authors suggest that reasoning about probabilities might appear much later in development, from around 5 years of age^7,8^.

The ability to make statistical inferences might be important for species other than humans, to make decisions in the face of uncertainty and/or to deal with unpredictable environments. To date, however, evidence of complex statistical skills in non-human animals (hereafter, animals) is extremely scant^9,10^. One reason for that is that statistical reasoning (i.e. predicting the probability of rewards based on the relative frequencies of objects^11^) can only be reliably demonstrated after ruling out alternative explanations based on simpler quantity heuristics (e.g. “select the container with a higher number of highly-liked food”, or “avoid the container with a higher number of less-preferred food”^12^).

Great apes, long-tailed macaques (*Macaca fascicularis*) and keas (*Nestor notabilis*) have shown statistical reasoning, using relative frequencies of items to predict sampling events^11,13–16^. Moreover, keas could combine information across different domains, integrating physical and social information when making statistical decisions^11^, in contrast to chimpanzees that succeeded in integrating social information, but failed to integrate physical information when predicting sampling outcomes^17^. In other species, evidence of statistical skills is yet missing, as individuals may have used simpler quantity heuristics to solve the task. Capuchin monkeys (*Sapajus apella*), for instance, successfully predicted sampling outcomes that could not be inferred by simply comparing the number of highly-liked items, but failed to do so when they could not simply avoid the container with a higher number of less-preferred items, thus suggesting that, at least in some contexts, capuchin monkeys use simpler quantity heuristics to make decisions^8^. Similarly, it is not clear yet whether rhesus monkeys (*Macaca mulatta*), African grey parrots (*Psittacus erithacus*) and pigeons (*Columba livia*) really use quantity heuristics or relative frequencies to predict sampling outcomes, as controls for the use of quantity heuristics are usually missing^18–20^; see^11^.

The fact that both primates and keas show evidence of statistical reasoning suggests that statistical skills can convergently evolve in different taxa, despite differences in brain structure and neural density^11,14,15,21^. Given that both primates and keas have brains with a large relative size^22,23^, however, also raises the question of whether large brain sizes are a necessary prerequisite for the emergence of complex statistical skills. Here, we tested this hypothesis by studying statistical reasoning in an ungulate species, giraffes (*Giraffa camelopardalis*). Giraffes are an ideal model for this study: they perform well in different tasks of physical cognition (e.g. object permanence^24^, memory^25^, quantity discrimination^26^), and are characterized by high fission–fusion levels^27,28^ and large dietary breadth^29^—two features that have been linked to the emergence of complex cognition^30,31^. Moreover, in contrast to primates and keas, giraffes have a relatively small brain size, with an encephalization quotient of 0.64^32^, which is quite small in comparison to the 2.48 of chimpanzees^22^ or the 1.42 of keas^23^. Therefore, giraffes may show complex cognitive skills as the result of specific selective pressures experienced in certain socio-ecological conditions, although they might not have especially large brains.

**Figure 1:**
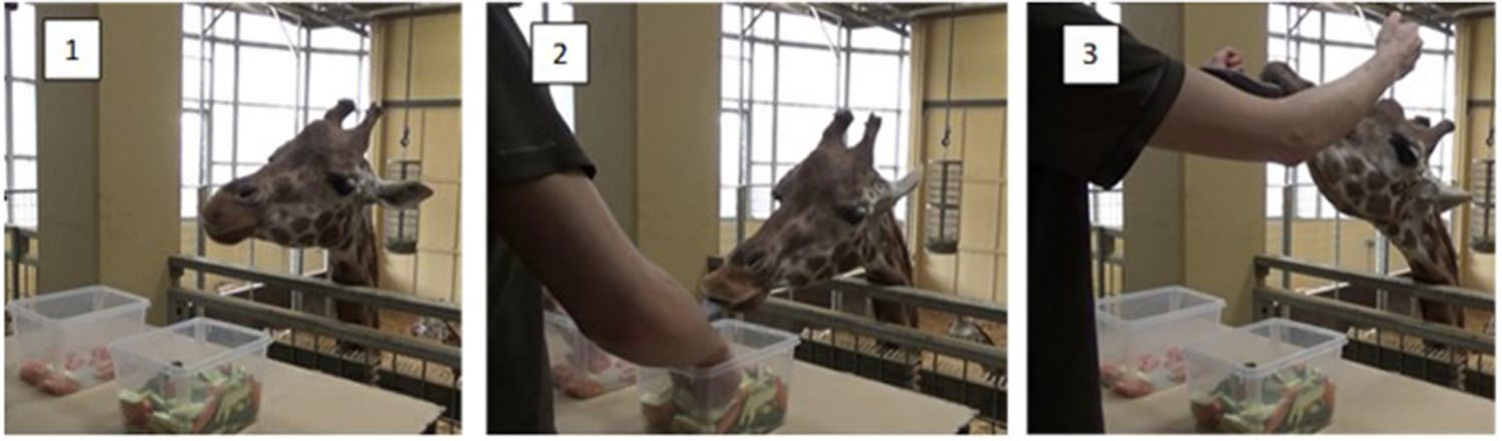
An example of a trial in Experiment 1, condition 2. Picture 1, the experimenter presents the two containers to the subject. Picture 2, the experimenter simultaneously takes one food piece from each container, without the giraffe seeing which piece is taken. Picture 3, the giraffe selects one of the two outcomes by touching it with the tongue.

In this study, we followed the procedure used by previous studies on this topic^5,11,14^. In Experiment 1, giraffes were presented with two transparent containers with different frequencies of highly liked (i.e. carrots) and less-preferred food (i.e. zucchini). The experimenter simultaneously took one piece from each container with his hands, without the giraffe seeing which piece was actually taken (Fig. 1). The giraffe could then select one of the two outcomes by touching one of the two closed fists. Task 2 was identical, except that the two food containers were divided in two parts by a physical barrier, so that only the food in the upper part of the containers was accessible to the experimenter and had to be accounted for while making decisions (see Video 1 in Supplementary Information). We hypothesized that, if large brains are necessary for the emergence of statistical skills, giraffes would not be able to make statistical inferences and combine information across different domains.

## Results

In the first experimental task, we tested if giraffes were able to make decisions based on the relative frequencies of food items in the containers. We included three different conditions aimed to rule out the use of simpler quantitative heuristics (Table 1). In condition 1, subjects were expected to preferentially choose the container with 100 carrots + 20 zucchinis over the one with 20 carrots + 100 zucchinis if they were comparing relative frequencies. In condition 2, we expected that subjects would prefer the container with 20 carrots + 4 zucchinis over the one with 20 carrots + 100 zucchinis. In Condition 3, subjects were expected to choose the container with 57 carrots + 63 zucchinis over the one with 3 carrots + 63 zucchinis. All study subjects (N = 4) could solve the three conditions of the first task already in the first session (i.e., 17 out of 20 trials selecting the expected container), except for one subject in condition 2 and one in condition 3, who required two sessions to solve the condition.

**Table 1:** Performance of the study subjects in all tasks and conditions (nominators represent the number of correct choices out of the total trials of each session, i.e. the number of trials in which subjects selected the carrot or, in C2, the food sampled from the container with a higher relative frequency of carrots).

Subjects	Sex	Task 1			Task 2		Controls		
		C1	C2	C3	C1	C2	C1	C2	C3
		100 + 20 20 + 100	20 + 100 20 + 4	3 + 63 57 + 63	20 + 4 (20 + 36) 20 + 20 (20 + 20)	20 + 20 (20 + 20) 4 + 20 (36 + 20)	–	100 + 20 20 + 100	20 + 100 20 + 4
Nakuru	M	17/20	18/20	18/20	9/20 + 15/20 + 11/20 + 2/20	–	8/12	13/20	11/12
Njano	M	17/20	17/20	3/20 + 17/20	10/20 + 2/20 + 1/20 + 8/20	–	7/12	16/20	10/12
Nuru	F	17/20	4/20 + 17/20	18/20	5/20 + 17/20	18/20	7/12	17/20	9/12
Yalinga	F	17/20	17/20	17/20	3/20 + 4/20 + 7/20 + 2/20	–	4/12	17/20	12/12

Below each condition (C) we report the different quantities presented to the animal (one line for each container: the first number of each line indicates the number of preferred food pieces, and the second one the number of less preferred food pieces). For task two we also report (in parenthesis) the number of food pieces below the partition.

The second experimental task was harder to master, because subjects had to also integrate physical information about the barrier internally dividing the container, in order to correctly predict sampling information (Table 1). Depending on the condition, we expected subjects to preferentially choose the container with 20 carrots + 4 zucchinis above the partition, and 20 carrots + 36 zucchinis below it (over the one with 20 carrots + 20 zucchinis above the partition, and 20 carrots + 20 zucchinis below it), and the one with 20 carrots + 20 zucchinis above the partition, and 20 carrots + 20 zucchinis below it (over the one with 4 carrots + 20 zucchinis above the partition, and 36 carrots + 20 zucchinis below it). In this task, three of the four subjects failed to pass the first condition after four sessions (Table 1). Only one subject passed the first condition (in the second session), and was therefore tested also in the second condition, which was solved in the first session.

Finally, we administered three control conditions to rule out that giraffes solved the task by using information other than relative frequencies (i.e. olfactory cues, inadvertent visual cues by the experimenter when sampling or holding the food, absolute quantities visible in the upper part of the containers; Table 1). In the first condition, giraffes could only rely on olfactory cues (but not on relative frequencies) to locate the carrot. In the second condition, giraffes relying on relative frequencies (rather than inadvertent visual cues by the experimenter) should have chosen the hand with the zucchini sampled from the container with a higher relative frequency of carrots. In the third condition, giraffes relying on relative frequencies (rather than visible absolute quantities) should have chosen the carrot sampled from the container with a higher relative frequency of carrots, although both containers were first presented with an equal number of carrots each.

We used Generalized Linear Mixed Models to assess whether the probability of making the correct choice (i.e. choosing the container more likely to provide the preferred food) differed across conditions and trials, when controlling for the side chosen. The full model significantly differed from the null one (GLMM: *χ*^2^ = 35.96, *df* = 15, *p* < 0.001). Condition had a significant effect as main term on the probability to choose the correct container (*p* < 0.001). In particular, with regards to the experimental conditions, subjects performed significantly worse in the first condition of the second task, than in the first and third conditions of the first task (vs. condition 1: *p* = 0.046; condition 3: *p* = 0.033). With regards to control conditions, subjects performed significantly worse in the first control condition (i.e. olfactory cues) than in most of the other conditions (vs. conditions 1, 2 and 3 of the first task: *p* = 0.005, *p* = 0.011 and *p* = 0.004, respectively; second control condition, i.e. inadvertent visual cues: *p* = 0.025). The second and third control conditions (i.e. inadvertent visual cues and visible absolute quantities, respectively), in contrast, did not significantly differ from any experimental condition (all *p* > 0.005). Finally, neither trial number (*p* = 0.910) nor side chosen (*p* = 0.315) had a significant effect on the response, suggesting no important learning effects and side biases in our study (Table 2).

**Table 2:** Results of the full model run, with estimates, standard errors (SE), confidence intervals (CIs) and p values for test predictors (significant values are marked with an asterisk).

Predictors and controls	Estimate	SE	2.5% to 97.5% CIs	*p*
Intercept	1.86	0.35	1.17 to 2.56	–
Task 1, condition 2	− 0.17	0.42	− 1.00 to 0.66	< 0.001*
Task 1, condition 3	0.00	0.43	− 0.85 to 0.85	
Task 2, condition 1	− 1.15	0.37	− 1.88 to − 0.41	
Task 2, condition 2	0.46	0.81	− 1.22 to 2.05	
Control condition 1	− 1.62	0.43	− 2.46 to − 0.77	
Control condition 2	− 0.43	0.41	− 1.24 to 0.39	
Control condition 3	0.03	0.51	− 0.98 to 1.04	
Trial number	0.00	0.01	− 0.02 to 0.02	0.910
Side chosen	− 0.20	0.20	− 0.60 to 0.19	0.315

## Discussion

In this study, giraffes could reliably make statistical inferences based on the relative frequencies of two different food types. Like chimpanzees and keas^11,14^, giraffes spontaneously selected the container more likely to provide the preferred food in the experimental conditions, even when subjects could not rely on simpler quantity heuristics (e.g. because the correct container did not contain a higher number of highly-liked food, and the wrong container did not contain a higher number of less-preferred food). The relative brain size of giraffes is small, and smaller than the one of keas and primates^22,23,32^, the only species for which statistical reasoning has been shown so far^11^. Therefore, these results suggest that large relative brain sizes are not a necessary prerequisite for the evolution of complex statistical skills, and that the ability to make statistical inferences may be widespread in the animal kingdom.

Giraffes were surprisingly fast at solving the first experimental task, requiring on average 1.2 sessions to reliably select the correct container in at least 17 out of 20 trials. In contrast, keas tested with the same procedure required an average of 3.9 sessions, and up to 11 sessions, to solve the task^11^. Although it is possible that the specific socio-ecological pressures faced by giraffes^27,28^ might be linked to the evolution of complex cognitive skills, including enhanced statistical abilities, it is also possible that the use of tokens might have made the procedure more complex for keas^33^; but see^34^. Compared to great apes and long-tailed macaques, giraffes showed a similar performance, but giraffes were administered more trials than the other species (e.g. 23 trials on average in the first task^12,13^). Although it is possible that this might have facilitated performance in giraffes, it should be noted that we detected no learning effects in our study.

In the second task, only one giraffe could successfully integrate physical information when making statistical inferences, suggesting that the physical barrier greatly increased the difficulty of the task. In contrast to the first task, keas appeared to be more proficient than giraffes, with five out of six individuals solving the task after an average of only 1.9 sessions^11^. Given that this task requires the ability to integrate information across multiple cognitive domains^11^, the lower performance of giraffes in this task might suggest that, whereas they can reliably make inferences based on the relative frequencies of objects, their ability to integrate information across cognitive domains may be more limited. However, one should also note that, in contrast to keas, our study subjects did not go through a training phase to acquire knowledge about the physical properties of the barriers in the container. Future studies should therefore explore whether giraffes really have a limited ability to integrate information across domains. Moreover, the fact that one individual reliably solved both conditions of the second task, without previous training and after no more than two sessions, suggests that at least some individuals may be able to spontaneously integrate information from different domains to make decisions under uncertainty.

Finally, several control conditions confirmed that giraffes really made their choices based on the relative frequencies of food in the containers, and not on other information. When giraffes could only rely on olfactory cues to locate the carrot (but not on relative frequencies), their performance significantly decreased, suggesting that the use of olfactory cues could not explain their successful performance in the experimental conditions. Moreover, when we sampled zucchini from the container with a higher relative frequency of carrots, giraffes still preferentially selected the container with a higher relative frequency of carrots. Finally, when an equal quantity of carrots was visible in both containers at the beginning of the trial, and then covered with zucchini, giraffes could still successfully solve the task, and their performance did not decrease from the one shown in the other experimental conditions. Overall, these results therefore suggest that the use of olfactory cues, inadvertent visual cues by the experimenter and the amount of visible absolute food quantities cannot explain the successful performance of the giraffes in the experimental conditions.

In evolutionary terms, statistical abilities might provide crucial fitness benefits to individuals when making inferences in a situation of uncertainty, and it should, therefore, not be surprising if these abilities are widespread across animal taxa. In the future, it would be interesting to test more species with these experimental procedures, and use a comparative approach to assess whether the specific socio-ecological challenges faced by different species reliably predict the distribution of statistical skills across animals. Very likely, statistical skills may be present in several other taxa.

## Methods

### Ethics

This research was approved and supervised by the staff of the Zoo of Barcelona. This study strictly adhered to the legal guidelines and regulations of the country in which it was conducted (Spain), and in accordance to the ARRIVE guidelines^35^. The study was considered a form of enrichment for the giraffes and no further permits were required.

### Subjects

Our study subjects were two male and two female giraffes (*Giraffa camelopardalis*) housed at the zoo of Barcelona. All study subjects were fed a regular diet of fruit and vegetables, and had limited experience with experimental tasks^19–21^. Participants were never food or water deprived during this study, and participation was on a completely voluntary basis. The individuals could approach the experimenter at any time to participate in the study.

### Experimental procedures

The procedure consisted of one food-preference task, two experimental tasks (for a total of 5 conditions), and three control conditions. In the food-preference task, we assessed individual food preferences by presenting each subject with two out-of-reach identical transparent containers, one with 120 pieces of zucchini and the other one with 120 pieces of carrots, all of the same size and form. We selected zucchini and carrots based on previous observations of the same subjects during a pilot study. In full view of the subject, the experimenter simultaneously put one hand in each of the two containers, grabbed one piece of food with each hand (making sure that the choice was not visible), and simultaneously presented the closed fists to the subject to make a choice. Subjects were tested in 20 trials and moved to the experimental tasks only if selecting the preferred food (i.e. carrots) in at least 17 trials. All individuals passed the criterion in the preference test.

**Figure 2:**
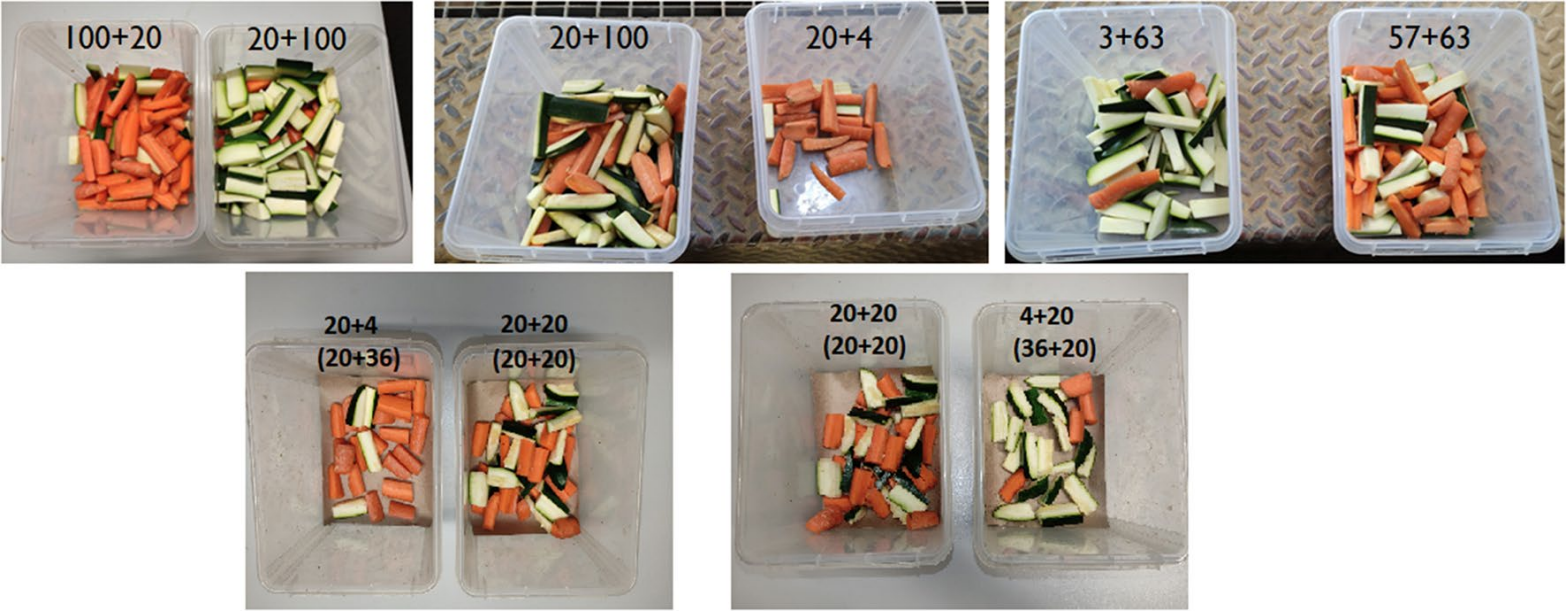
A picture with the stimuli used in each condition of Experiment 1 (from left to right: condition 1, condition 2 and condition 3).

The two experimental tasks largely followed the procedures used by Bastos and Taylor^11^, but we reduced all training phases to minimize learning effects. The first experimental task consisted of three conditions, aimed to assess whether subjects could reliably select the preferred food based on the relative frequencies of the two food types, rather than on the absolute quantities presented (see Fig. 2). In the first condition, the procedure was identical to the food-preference task, but the two containers had 20 carrots + 100 zucchinis, and 100 carrots + 20 zucchinis, respectively. In the second condition, the two containers had 20 carrots + 100 zucchinis, and 20 carrots + 4 zucchinis. In this condition, we predicted that giraffes would preferentially select the second container if comparing relative frequencies, but show no preference if comparing absolute quantities of the preferred food. In the third condition, the two containers had 57 carrots + 63 zucchinis, and 3 carrots + 63 zucchinis. As above, we predicted that giraffes would preferentially select the first container if comparing relative frequencies, but show no preference if comparing absolute quantities of the less-preferred food. In each condition, subjects could obtain the preferred food by comparing relative frequencies and selecting the container more likely to provide carrots (i.e. 100 carrots + 20 zucchinis, 20 carrots + 4 zucchinis, and 57 carrots + 63 zucchinis, respectively). If they did so in at least 17 out of 20 consecutive trials, they proceeded to the next condition, otherwise they received another session of the same condition, up to a maximum of 4 sessions (see Supplementary Information for a Video Example).

In the second experimental task, we tested whether giraffes can integrate physical information when making statistical inferences. We followed the same procedure as in the previous task. In the first condition, both containers had 40 carrots + 40 zucchinis. However, both containers were internally divided by a horizontal partition, so that only the food rewards above the partition could be sampled by the experimenter, and giraffes had to make their decision by only assessing the content in the upper part of the containers. Following Bastos and Taylor^11^, we presented individuals with a container with 20 carrots + 20 zucchinis above the partition, and 20 carrots + 20 zucchinis below the partition, and with a second container with 20 carrots + 4 zucchinis above the partition, and 20 carrots + 36 zucchinis below it. We predicted that, if giraffes could also use their understanding of physical barriers when making statistical inferences, they should have preferentially selected the second container. The second condition was identical, but the number of carrots and zucchinis was inverted. In all tasks and conditions, we pseudo-randomized and counterbalanced the side of each container across trials. In both experiments, the experimenter always drew from the containers a piece of food belonging to the majority food type, as in previous studies^11,14^.

Finally, we ran three control conditions to rule out alternative explanations based on simpler quantity heuristics and learning processes. First, we ran a condition to determine if giraffes relied on olfactory cues rather than relative frequencies to locate carrots. The procedure was similar to the food-preference task, but subjects did not see the containers from which food was sampled from. They had to choose between the two closed fists without watching which piece of food the experimenter held in each hand. Subjects were expected to be successful in this condition (i.e. selecting the hand with the carrot) if they relied on olfactory cues to locate the food, but not if they relied on their vision, as they could not see which piece of food the experimenter had sampled. Second, we ran a condition to rule out the possibility that the experimenter inadvertently provided visual cues to the giraffes when sampling the food and/or holding the food in the hands. We followed the same procedure as in the first condition of the first experimental task, but the experimenter always retrieved from the containers the least probable piece of food for that population. Subjects were expected to be successful in this condition (i.e. selecting the food sampled from the container with a higher relative frequency of carrots, which in this case were zucchini) if they relied on the relative frequencies of food, but not if they relied on inadvertent visual cues provided by the experimenter during the sampling procedure (in which case, subjects should have preferentially selected the container with a lower relative frequency of carrots, from which carrots were sampled). Third, we ran a condition to determine if giraffes relied on the overall absolute quantity of visible preferred food (as this food was partially covering the less-preferred food, and could have been visually more salient) rather than relative frequencies of the food in the containers. We followed the same procedure as in the second condition of the first experimental task, but the experimenter first showed the containers with only the carrots (having identical absolute quantities), and then added the zucchini in both containers, in full view of the subject. Subjects were expected to be successful in this condition (i.e. selecting the hand with the carrot sampled from the container with a higher relative frequency of carrots) if they relied on the relative frequencies of food, but not if they relied on the absolute quantities visible in the upper part of the containers (in which case, performance should have dropped at chance levels). For each of the three control conditions, we respectively ran 12 trials, 20 trials and 12 trials for each individual. We ran less trials for the olfactory condition because we had already tested this in previous experiments with negative results^24^. In the third control condition, we ran less trials due to time constraints.

### Statistical analyses

We assessed individual performance in each experimental condition as the number of trials in which the subject made the correct choice (i.e. selecting the carrot in the first control condition C1; selecting the zucchini sampled from the container with a higher relative frequency of carrots in the second control condition C2; and selecting the carrot sampled from the container with a higher relative frequency of carrots in all the other conditions; see Table 1). To compare performance across conditions, and assess possible learning effects, we further run a generalized linear mixed model^36^ in R (R Core Team, version 4.0.1), using the “glmmTMB” package^37^. We used a binomial distribution to assess whether the probability to make the correct choice varied across trials and conditions, when controlling for the side chosen, including subject identity as random factor. Trial number and condition were first entered in interaction as test predictors, and then only entered as main effects as the interaction term was not significant. This final model was then compared to a null model which only included controls and random effects, using likelihood ratio tests^38^. In case of significant categorical test predictors (i.e. condition), we ran post-hoc tests with Tukey corrections to compare the different levels of the predictor. We detected no problems when checking residual diagnostics and overdispersion using the “DHARMa” package^39^.We further checked multicollinearity with the “performance” package^40^, which was no issue (maximum variance inflation factors = 1.40^41^).

## Supplementary Information

Supplementary Information 1.

Supplementary Information 2.

Supplementary Legends.

Supplementary Video 1.

## Data Availability

Our dataset and code are available in Supplementary Information.
