# Supplementary material for: Giraffes make decisions based on statistical information
Source: Sci Rep. 2023 May 4;13:5558. doi: 10.1038/s41598-023-32615-3 (PMC10160108; doi:10.1038/s41598-023-32615-3)
Supplement: Supplementary file 3 — Supplementary Legends. [file 41598_2023_32615_MOESM3_ESM.docx]

**Video example of each experiment**

A trial of each Experiment and a trial from the point of view of the giraffe.
